# Optimization of fracturing technology for unconventional dense oil reservoirs based on rock brittleness index

**DOI:** 10.1038/s41598-024-66114-w

**Published:** 2024-07-02

**Authors:** Huimei Wu, Nan Zhang, Yishan Lou, Xiaopeng Zhai, Bin Liu, Song Li

**Affiliations:** 1 National Engineering Research Center for Oil and Gas Drilling and Completion Technology, Yangtze University, Wuhan, 430100 China; 2 Hubei Key Laboratory of Oil and Gas Drilling and Production Engineering, Yangtze University, Wuhan, 430100 China; 3 Turpan Oil Production Plant, PetroChina Tuha Oilfield Company, Hami, 839000 China; 4 School of Earth Sciences, Northeast Petroleum University, Daqing, 163318 China

**Keywords:** Tight oil reservoir, Volume fracturing, Adaptability, Brittleness index, Fracturing simulation, Energy science and technology, Engineering

## Abstract

The concept of volume fracturing has revolutionized the conventional limits of low permeability, expanded the effective resource space, and significantly enhanced oil well production in tight oil reservoir development. This paper elucidates the mechanism of volume fracturing technology for tight sandstone reservoirs by considering multiple factors such as the initiation range of multi-fractures, influence of far-well horizontal principal stress on fracture initiation and propagation, degree of natural fractures development, and mechanical parameters of reservoir rock. Through simulation based on the mechanical parameters of reservoir rock, a comparative analysis was conducted between the model-calculated rock fracture pressure value and measured data from fracturing construction wells in the study area. The results revealed that there was a discrepancy within 10% between the model calculations and actual data. By simulating the effects of different injection volumes of fracturing fluid, pumping rates, and perforation methods on the fracture geometry, optimal design parameters for volume fracturing technology were obtained. Additionally, we propose optimization ideas and suggestions for construction parameters applicable to field operations. The simulation results indicate that a minimum recommended fluid volume scale exceeding 1800 m^3^ is advised for the reservoir. Based on frictional calculations, it is recommended to have an on-site construction rate not less than 18.0 m^3^/min along with 36–48 holes/section for perforation purposes. The numerical simulation research presented in this paper provides a theoretical reference basis and practical guidance for the application of fracturing network technology in tight sandstone reservoirs.

## Introduction

The introduction and advancement of network fracturing concepts in the development of tight oil reservoirs have lowered the threshold for low permeability, broadened the space of available resources, and significantly boosted oil well production^1–5^. The advancement of multi-stage fracturing technology for horizontal wells and multi-layer fracturing technology for vertical wells is pivotal to the effective development of global tight oil and gas resources^6,7^. In recent years, as domestic horizontal well technology has matured, the proportion of horizontal wells used to develop low-permeability tight oil reservoirs has gradually increased. We have gained valuable experience in exploring and developing tight oil reservoirs while making significant progress on supporting process technologies^8,9^. Successful development provides valuable insights into unconventional tight oils with similar reservoir conditions, from which corresponding fracturing parameters can be derived. Achieving the anticipated production increase in these reservoirs may prove unattainable through the utilization of a solitary primary fracture, particularly when considering the inherent heterogeneity and anisotropy of the rock formation. A more comprehensive understanding of the reservoir’s properties and the interplay between fractures is essential for formulating an effective stimulation strategy.

The initiation and propagation of multiple fractures within the well are crucial factors in network fracturing. Since 2006, numerous domestic scholars have conducted extensive research on the formation mechanism and operational conditions of multiple fractures^10–12^. Meanwhile, researchers have summarized the fundamental principles of network fracturing from various perspectives, including rock mechanics, simulation experiments, and mathematical calculation models^13–16^. In ultra-low permeability reservoirs, it can be quite challenging to depend solely on a single main fracture to achieve the expected production increase. This is primarily due to the nature of these reservoirs, which possess extremely low flow rates and require intricate stimulation techniques to enhance their productivity. These reservoirs often contain multiple fractures, each with unique properties and characteristics^17^. Therefore, relying solely on a single primary fracture may not be sufficient to fully unlock the reservoir’s potential. It is crucial to consider the interconnectedness of these fractures and their interactions in facilitating fluid flow^18,19^. Furthermore, the complexity of these reservoirs necessitates the utilization of advanced technologies and integrated approaches to effectively stimulate the target fractures and enhance reservoir performance, which may involve hydraulic fracturing, proppant injection, and advanced monitoring techniques for optimizing the stimulation process.

Numerical simulation technology is a crucial solution for accurately replicating fracturing initiation and propagation behaviors, compensating for limited experimentation in large-scale rock formations^20,21^. It allows convenient adjustments of various parameters to investigate the underlying principles governing fracture development under diverse conditions, significantly enhancing our comprehension of rock fracturing mechanisms while optimizing fracture design^22–24^. Alekseenko et al.^25^ established a three-dimensional fracture initiation numerical model in linear elastic strata, which can be used to determine the fracture initiation pressure, initiation location, and initiation direction. After analyzing the effects of multiple stages of fracturing, it was found that the angle between the perforation hole and the fracture surface is a significant factor affecting fracture initiation pressure^25^. Additionally, considering the influence of in-situ stress, the change in fracture initiation pressure is primarily dependent on perforation parameters and wellbore orientation. Wu et al.^26^ proposed considering the complex fracture system resulting from shale deformation as a dual medium model. By integrating productivity data with micro seismic data, they established a fracture network model^26^. Bazan et al.^27^ proposed a method for evaluating the pressure loss at the wellbore and near-wellbore perforation holes, determining cluster efficiency, assessing divertor performance, analyzing fracturing construction pressure, and using segment construction data to analyze the real-time fracture geometry. This method clarifies the influence of factors such as the size and number of perforation holes, cluster spacing, divertor application, and stress shadow on the resulting fractures. Ahmad et al.^28^ utilized a fully coupled poroelastic model to assess the impact of poroelastic mechanisms on stimulation volume, repeated fracturing, and fracture clusters. The simulation results indicated that rock fractures are more likely to occur in the vicinity of fractures, particularly near the fracture tips, and there’s a possibility of shear failure at locations distant from the fracture walls^28^. When hydraulic fractures are subjected to pressure, the coupling of poroelastic effects is unlikely to lead to the opening and expansion of multiple hydraulic fractures, resulting in the formation of so-called fracture clusters at a certain distance from the hydraulic fracture surface^19,29^. Li et al.^30^ introduced an effective comprehensive hydraulic fracture height growth model based on a simplified three-dimensional displacement discontinuity method and incorporated it into the hydraulic fracture simulator to investigate the influence of weak boundaries on the geometry and width distribution of hydraulic fractures. The findings suggest that the presence of weak boundaries significantly impacts the distribution of fracture height, lateral length, and width. Weak interfaces lead to a decrease in hydraulic fracture height vertically, resulting in an elongated lateral length and reduced overall width of the fracture. The spacing and shear toughness of these weak interfaces are crucial factors in determining the geometry and width values of fractures. In cases where multiple fractures occur, weak interfaces influence external fractures by reducing average height and total width distribution while increasing lateral length.

The volume fracturing technique not only considers the compressibility of the formation and the initiation and extension behavior of fractures in the near-wellbore zone, but also accounts for the influence of natural fractures in the far-wellbore zone on fracture propagation. This paper assesses the applicability of volume fracturing technology for tight oil reservoirs in this study area based on brittleness index and reservoir rock mechanical properties, guided by specific geological and rock mechanical characteristics. Additionally, it investigates fracture initiation in the near-wellbore zone and the influence of natural fractures in the far-wellbore zone on fracture extension. Based on these findings, specific optimization concepts are proposed for designing parameters for single well network fracturing, offering theoretical references and practical guidance for implementing complex network fracturing technology in this study area.

## Characteristics of block reservoirs

### Petrology characteristics of formation

The reservoir in the study area primarily consists of a sequence of light grey to grey–green feldspar fine sandstone, followed by silty to fine feldspar sandstone and medium to fine feldspar sandstone. Detrital particles make up approximately 81% of the composition, predominantly composed of feldspar, followed by quartz, mica, and a small amount of barite. Potash feldspar and acid plagioclase are the main components with content ranging from 37 to 57%, averaging at 45.4%. Metamorphic rock detritus accounts for around 18–36% (average: 25.7%), mainly consisting of metamorphic rock fragments, along with an additional presence of detrital materials ranging from 5 to 10% (average: 7.5%).

### Reservoir porosity, permeability, and natural fracture characteristics

The porosity distribution of the reservoir ranges from 0.72 to 16.3%, with an average value of 7.98%, and the permeability distribution ranges from 0.002 to 20.89 mD, with an average value of 0.87 mD.

The extent of natural fracture development plays a pivotal role in determining the efficacy of fracturing within a fracture network. When natural fractures are highly developed, they have an increased likelihood of inducing hydraulic fractures at multiple stages and intervals, resulting in the formation of a complex network structure. In our study area, the density of natural fractures consistently exceeds 2 per square meter and generally exhibits a high degree of development, which facilitates the formation of intricate hydraulic fracture networks.

The porosity of fractures in reservoir is approximately 1.8–2.7%, primarily existing in two forms: structural fractures that intersect at high angles or nearly vertically cut through deposited grains and cements, with most remaining unfilled and occasionally containing bituminous fillings. Conversely, diagenetic fractures are formed by sliding movements in soft sediments such as biotite during compaction due to uneven stress.

## Methodology

### Research methodology

**Figure 1:**
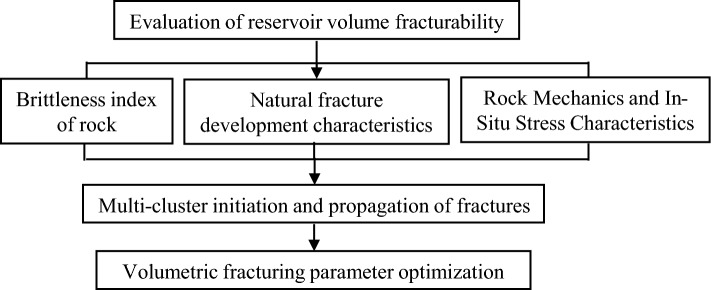
Research technical route.

Based on the aforementioned analysis of reservoir geological characteristics, we initially conducted an in-depth investigation into the brittleness characteristics of reservoir rocks and discussed their impact on the process of volume fracturing. Building upon this foundation, we further comprehensively analyzed rock mechanics, in-situ stress, as well as natural fracture development to gain a comprehensive understanding of these factors’ primary control effect on volume fracturing. Through a comprehensive evaluation, we accurately assessed the feasibility of creating a complex fracture network using volume fracturing and provided an essential theoretical basis for subsequent volume fracturing design. By utilizing numerical simulation technology to optimize the parameters involved in volume fracturing simulations, our aim was to maximize its effectiveness. In order to enhance the specificity and practicality of our numerical simulation design for volume fracturing, we conducted extensive simulation experiments considering various possible parameter combinations to identify the optimal configuration. This contributed to the enrichment of our theoretical knowledge and provided guidance for practical operations. Finally, based on researched theories and methods (Fig. 1), a highly practical and targeted set of numerical simulation technology was developed specifically to address the geological characteristics such as reservoir rock brittleness, rock mechanics properties including in-situ stress analysis, and natural fracture development within our study block; thereby facilitating the effective application of volumetric hydraulic fracturing numerical simulation technology.

### The initiation range of multiple fractures in perforated wells

The complex fracture network of volumetric fracturing refers to the simultaneous initiation and propagation of multiple radial multi-fractures along the wellbore, forming an interconnected system of fractures that extend independently from each other. This intricate fracture network is formed through the combined effects of two dynamic processes: multi-fracture initiation and propagation within the wellbore^31,32^. The gradual reduction in pressure difference between different directions within the wellbore facilitates the simultaneous initiation of multiple fractures, while the gradual increase in bottom hole fluid pressure plays a crucial role in promoting their concurrent propagation.

The fracturing well is completed through the process of perforation, while taking into account the calculation formula for fracturing pressure that considers filtration loss of fracturing fluid into the formation.

1 $$P_{f} = \frac{{3\sigma_{h} - \sigma_{v} + \sigma_{t} - \alpha \left( {\frac{1 - 2\nu }{{1 - \nu }}} \right)P_{p} }}{{1 + \phi - \alpha \left( {\frac{1 - 2\nu }{{1 - \nu }}} \right)P_{p} }}$$

where $P_{f}$ is the formation fracture pressure, MPa. $\sigma_{h}$ is the minimum horizontal principal stress, MPa. $\sigma_{t}$ is the rock tensile stress, MPa. $\sigma_{v}$ is the vertical principal stress, MPa. $\alpha$ is the Biot coefficient of rock, dimensionless. $\nu$ is Poisson’s ratio of rocks, dimensionless. $P_{p}$ is the formation pressure, MPa. $\phi$ is the formation porosity, %.

The fracture pressure can be calculated using the aforementioned formula and applied to Eq. (2) to determine the tangential and radial stresses of the well wall under the combined influence of various components. Considering the low tensile strength of rock, particular attention should be given to the tangential stress exerted on the wellbore^33^.

2 $$\left\{ {\begin{array}{*{20}l} {\sigma_{r} = P_{w} - \delta \phi \left( {P_{w} - P_{p} } \right)} \hfill \\ {\sigma_{\theta } = - P_{w} + \delta \left[ {\frac{{\alpha \left( {1 - 2\nu } \right)}}{1 - \nu } - \phi } \right]\left( {P_{w} - P_{p} } \right) + \sigma_{xx} \left( {1 - 2\cos 2\theta } \right) + \sigma_{yy} \left( {1 + 2\cos 2\theta } \right) - 4\tau_{xy} \sin 2\theta } \hfill \\ {\sigma_{z} = \delta \left[ {\frac{{\alpha \left( {1 - 2\nu } \right)}}{1 - \nu } - \phi } \right]\left( {P_{w} - P_{p} } \right) + \sigma_{zz} - \nu \left[ {2\left( {\sigma_{xx} - \sigma_{yy} } \right)\cos 2\theta + 4\tau_{xy} \sin 2\theta } \right]} \hfill \\ {\tau_{r\theta } = 0} \hfill \\ {\tau_{rz} = 0} \hfill \\ {\tau_{\theta z} = - 2\tau_{xz} + 2\tau_{yz} \cos \theta } \hfill \\ \end{array} } \right.$$

where $\sigma_{r}$, $\sigma_{\theta }$, $\sigma_{z}$ are the normal stress components in polar scale system respectively, MPa. $\sigma_{xx}$, $\sigma_{yy}$, $\sigma_{zz}$ are the normal stress components in in the coordinate system (x, y, z) respectively, MPa. $\tau_{r\theta }$, $\tau_{rz}$, $\tau_{\theta z}$ are the shear stress components in polar scale system respectively, MPa. $P_{w}$ is the bottomhole pressure, MPa. $P_{p}$ is the formational pressure, MPa. $\theta$ is the perforation azimuth, °.

According to the permeability well wall fracture criterion: $\sigma_{min} - \alpha P_{p} = - T$, the conditions required for well wall fracture initiation can be derived.

Where $\sigma_{min}$ is minimum tensile stress, MPa. $\alpha$ is the Biot coefficient of rock, dimensionless. $T$ is the tensile strength of rock, MPa.

**Figure 2:**
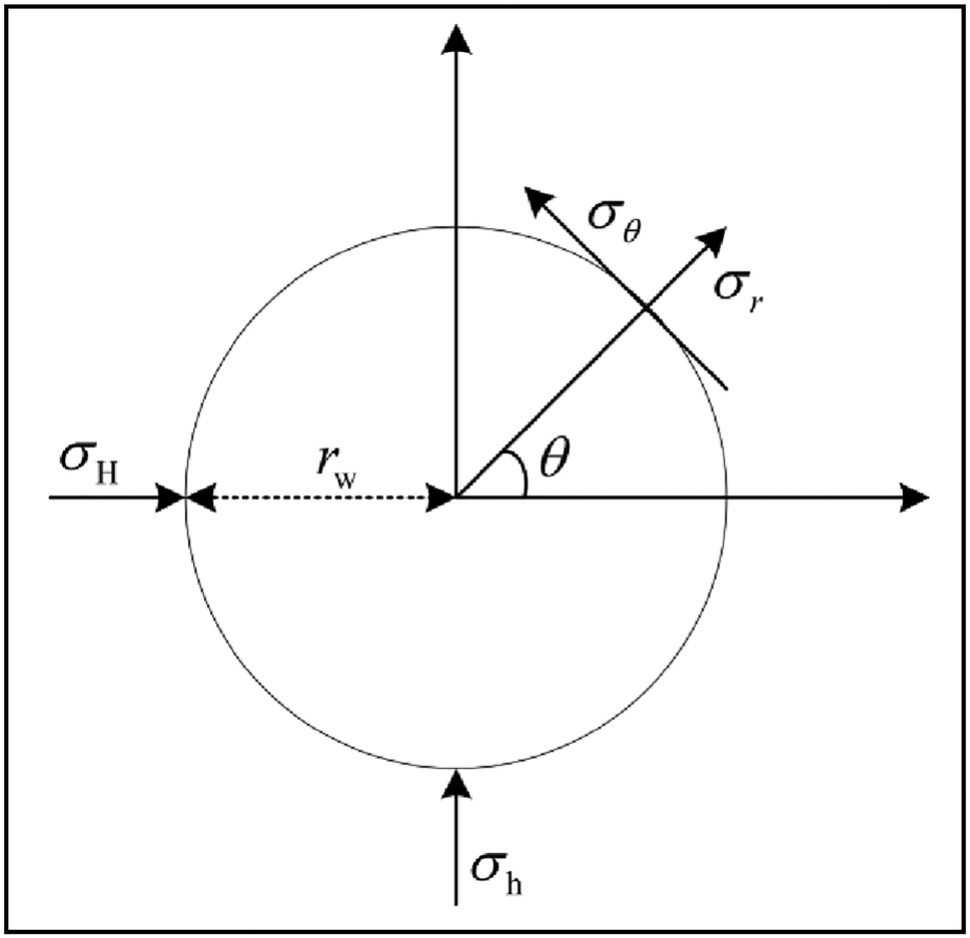
The distribution of the in-situ stress at the wellbore^34^.

The effectiveness of fracturing is directly influenced by perforation parameters, as demonstrated by both laboratory experiments and field applications. The distribution of in-situ stress after perforation was examined using the theory of elastic mechanics (Fig. 2). The findings revealed that the width of fractures is impacted by the orientation, density, and diameter of the perforations. An optimal perforation orientation exists based on specific formation and wellbore conditions. Therefore, optimizing the fracturing efficacy of perforated wells requires integrating the optimization of both perforation parameters and fracturing parameters.

**Figure 3:**
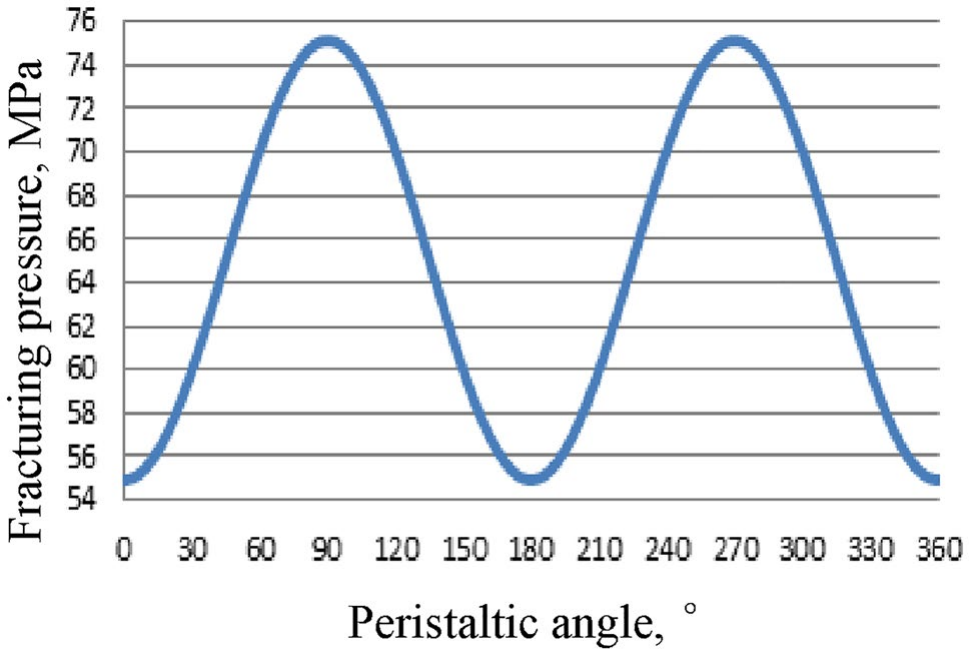
Prediction results of tangential force distribution around the well.

In Fig. 3, the abscissa represents the angle formed by the line connecting a point on the circumference of the wellbore to its center and the maximum horizontal principal stress, while the ordinate denotes tangential stress on the wellbore wall. If the wellhead pressure is limited to 100 MPa and maximum pumping displacement during fracturing stimulation is restricted to 18.0 m^3^/min, it is crucial that the maximum stress on the wellbore wall does not exceed 70 MPa; otherwise, fracture production becomes challenging. Analysis of Fig. 2 reveals that points along the circumference of the wellbore where near-bore initiation of fractures occurs easily are those with angle measures at either 0° or 180°.

### Horizontal principal stress analysis

The path of hydraulic fractures extending away from the wellbore is primarily determined by the distribution of the far-wellbore in-situ stress field. One of the most influential factors impacting fracture network formation is the disparity in in-situ stress levels. If this difference is excessively large, it becomes challenging to establish a network of fractures with significant dimensions; even then, only simple double-wing fractures can develop. Therefore, studying the in-situ stress conditions of individual wells is crucial. The distribution of the in-situ stress field can be calculated using commonly employed formulas for determining maximum and minimum horizontal principal stresses.

3 $$\left\{ {\begin{array}{*{20}c} {\sigma_{h} = \left( {\frac{\nu }{1 - \nu } + \beta_{1} } \right) \times \left( {\sigma_{v} - \alpha P_{p} } \right) + \alpha P_{p} } \\ {\sigma_{H} = \left( {\frac{\nu }{1 - \nu } + \beta_{2} } \right) \times \left( {\sigma_{v} - \alpha P_{p} } \right) + \alpha P_{p} } \\ \end{array} } \right.$$

where $\alpha$, $\beta$ are coefficients related to the actual reservoir block, which are obtained from previous fracturing data. $P_{p}$ is the formation pore pressure, MPa. $\nu$ is the Poisson’s ratio of the rock, dimensionless.

After analyzing over 40 wells, it has been determined that the stress differential in the region typically ranges from 3.5 to 8.7 MPa, indicating a relatively low magnitude. Consequently, it can be inferred that volumetric fracturing demonstrates high feasibility.

### Degree of natural fracture development

A critical factor in matrix fracturing is the extent of natural fracture development in close proximity to the wellbore. The more development these pre-existing fractures are, the higher their susceptibility to being induced by multiple stages fracturing, resulting in extension and ultimately leading to the formation of a complex network of fractures. The appearance of rock cores extracted from underground reservoirs was described, and the natural fracture development characteristics of the target layer were analyzed using statistical methods (Table 1). It is evident that the density of naturally occurring fractures in the vicinity of the well consistently exceeds 2.0/m, indicating a significantly increased level of fracture development throughout the block. This substantial degree of development greatly facilitates the creation of intricate fracture networks through hydraulic fracturing^35,36^.

**Table 1:** Statistics of natural fracture development in single wells in the study area.

Well number	Number of fractures	Density of fracture (/m)	Fracture width classification			Average fracture width (mm)
			Large	Moderate	Small	
H1	5	2.35		2	3	1.10
	13	3.79	3	2	8	1.22
H2	14	5.53		2	12	0.65
	17	3.42		3	14	0.63
H3	16	4.03		5	11	1.07
	23	4.57		11	12	0.87
H4	5	1.73		3	2	1.42
	10	4.62	2	8		2.31
Total amount	81	3.87	4	26	52	1.22

### Brittleness index of rock

Rickman et al.^37^ introduced a rapid assessment method for rock brittleness that can be directly applied on-site.

4 $$B_{Rick} = \frac{{\left( {E_{G} + \nu_{G} } \right)}}{2}$$

5 $$\left\{ \begin{gathered} E_{G} = \frac{{100(E - E_{\min } )}}{{E_{\max } - E_{\min } }} \hfill \\ \nu_{G} = \frac{{100(\nu - \nu_{\max } )}}{{\nu_{\min } - \nu_{\max } }} \hfill \\ \end{gathered} \right.$$

where $E_{G}$ is normalized Young’s modulus, dimensionless. $\nu_{G}$ is normalized Poisson’s ratio, dimensionless. $E_{min}$, $E_{max}$ are the minimum and maximum values of Young’s modulus in the block, GPa. $\nu_{min}$, $\nu_{max}$ are the minimum and maximum values of Poisson’s ratio in the block, dimensionless. $B_{Rick}$ is the Richman brittleness index.

**Figure 4:**
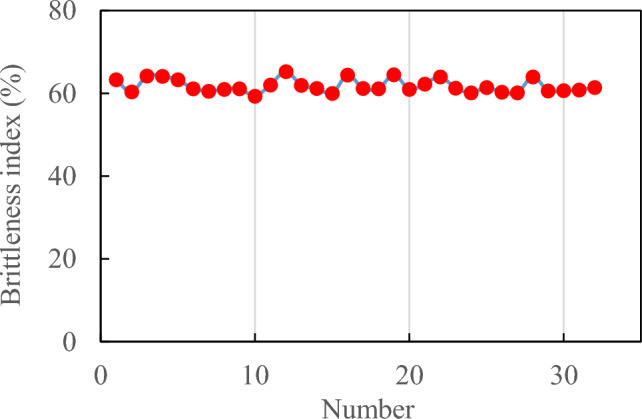
Brittleness index calculated based on rock mechanics parameters.

The high rock brittleness characteristic parameters result in the extensive development of a fracture network within the reservoir, necessitating an increase in the volume of fracturing fluid. However, it is possible to reduce both the concentration and quantity of proppant used. By integrating logging data from over 30 oil wells in the X well block and applying Eqs. (4) and (5), the brittleness index of the fracturing layers in these oil wells can be calculated. According to the calculation results, shown in Fig. 4, the majority of these wells exhibit a rock brittleness index ranging between around 60%, which promotes the formation of multiple fractures within the reservoir, facilitating a networked extension of hydraulic fractures.

The higher the brittleness index of rock, the greater the likelihood of rock fracture occurrence and the more developed the resulting fracture network becomes ^38^. When the brittleness index of rock falls within 10–20%, two-sided symmetrical fractures are prone to form. When the brittleness index ranges from 30 to 40%, multiple fractures tend to develop. When the brittleness index is between 40 and 50%, there is a transition in fracture morphology from a fracture network to multiple fractures. When the brittleness index exceeds 50%, pressure-generated fractures exhibit a network structure. Based on this analysis, it can be inferred that volume fracturing in the study area’s reservoir is likely to result in a significant number of complex and intricate fracture networks.

## Results and discussion

### Verification of model calculation results

The calculation formula for the formation fracture pressure error of the model is as follows.

6 $${\text{E}}rror = \left| {\frac{{P_{fc} - P_{fa} }}{{P_{fc} }}} \right|$$

7 $$P_{fa} = P_{pumping} + P_{fcp} - f - P_{p}$$

where $P_{fc}$ is the fracturing pressure of rock strata calculated by the model, MPa. $P_{fa}$ is the fracturing pressure in the actual hydraulic fracturing process of the reservoir, MPa. $P_{pumping}$ is the maximum surface pump pressure during the fracturing process, MPa. $P_{fcp}$ is the fluid column pressure, MPa. *f* is the frictional resistance generated by the fracturing fluid flowing through the tubing, MPa.

**Figure 5:**
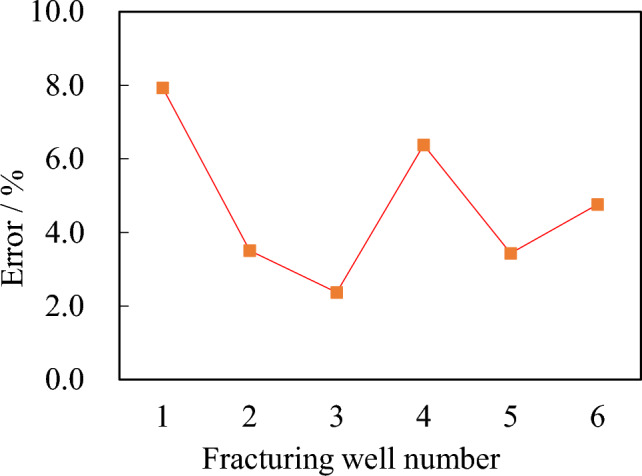
Error analysis of fracturing pressure value of fracturing well and model calculation results.

After analyzing the fracturing data of 6 wells in research area, Fig. 5 illustrates the error analysis between the calculated fracturing pressure results and the observed values in the study area. According to statistical analysis, there is an error range of 2.37–7.93% between the actual formation fracturing pressure and the model calculation results. Further verification revealed that all 6 wells’ fracturing pressure calculations were within a 10% margin, thus providing strong confirmation of the accuracy of our model calculations.

### Optimization of fracturing pumping displacement

The high density of fractures in the reservoir creates optimal conditions for the formation of intricate and reticulated fractures, but also results in rapid fluid loss and challenges in achieving wellbore pressure buildup. To address this issue, high-rate injection is necessary to swiftly establish pressure within the wellbore, facilitating simultaneous extension of multiple fractures. However, high-rate injection leads to increased friction which requires calculation for the fracturing string of a single well in the designated area as presented in Table 2.

**Table 2:** Fracturing string friction calculation.

Pumping displacement, m^3^/min		10	12	14	16	18	20
Friction coefficient, MPa/100 m	Tubing	0.93	1.71	1.98	2.67	3.31	3.85
	Casing pipe	0.22	0.27	0.36	0.60	0.89	1.23

The calculation assumes a tubing diameter of 88.9 mm and a casing diameter of 139.7 mm. The fracturing section is situated at a depth of 2150 m. According to the calculation, the friction in the casing is significantly lower than that in the tubing during injection. This holds particular significance in high in-situ stress areas, as excessive friction can lead to inadequate bottom-hole initiation pressure. This impedes the fracturing of the rock at the well’s bottom by preventing it from being broken open. Therefore, it is recommended to primarily employ casing injection on-site, especially for operations involving pumping displacement exceeding 18.0 m^3^/min.

**Figure 6:**
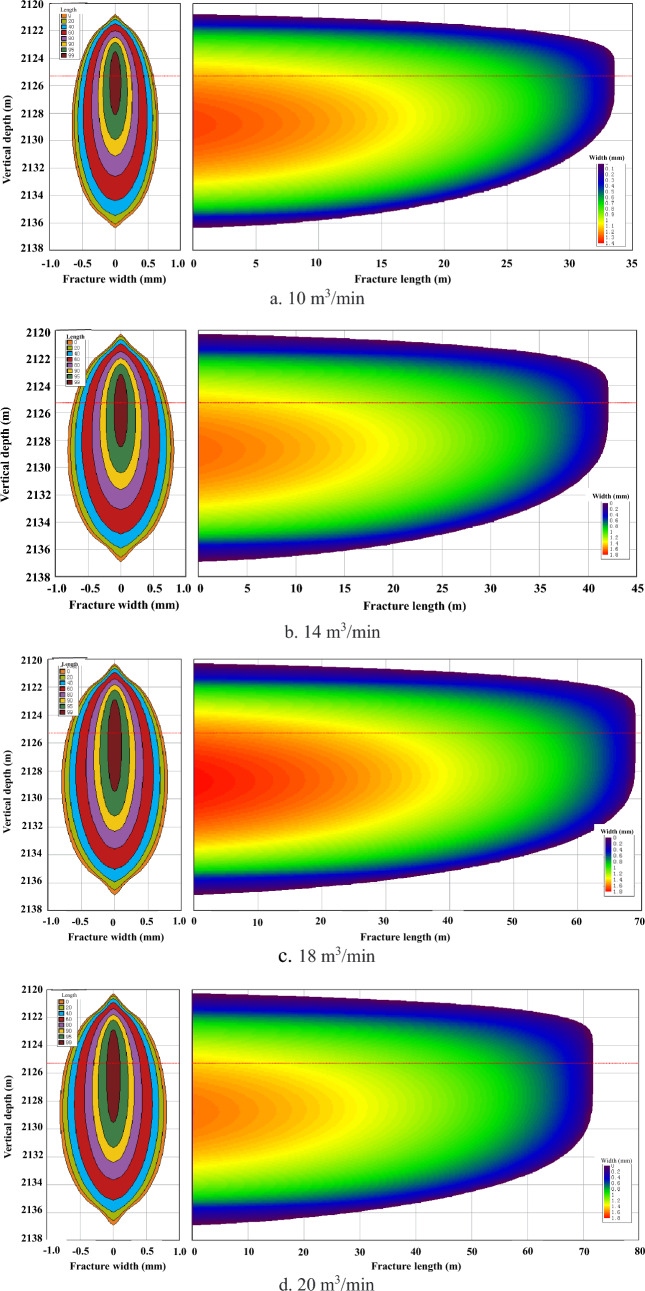
Fracture morphology under different pumping displacement of fracturing fluid.

In accordance with the wellbore storage effect theory, increasing the pumping displacement proves beneficial in effectively mitigating wellbore pressure by promoting higher net pressure and facilitating extensive three-dimensional fracture propagation. Furthermore, a larger pumping displacement becomes imperative for expanding fractures while minimizing filtration losses and optimizing fracturing fluid efficiency. Additionally, as a result of accelerated injection velocity of sand-laden fluids leading to reduced proppant sedimentation time and viscosity degradation-induced shorter pumping duration; substantial increase in pumping displacement significantly augments sand-carrying capacity as well. Nevertheless, it should be noted that there exists an optimal threshold for determining appropriate pumping displacements based on fracturing scale considerations. By setting the pumping displacement at 10–20 m^3^/min, simulation of fracture propagation was conducted to define the impact of each pumping displacement on fracture propagation. The calculation results are presented in Fig. 6a–d.

**Figure 7:**
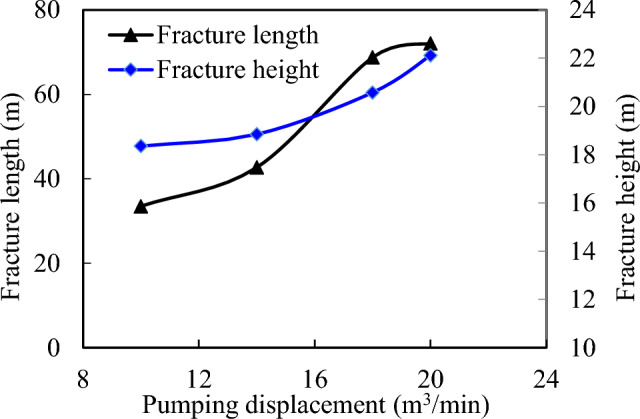
Influence of pumping displacement on fracture morphology.

The data illustrated in Fig. 6 demonstrates that with an increase in displacement up to 18 m^3^/min, there is an evident deceleration observed in the growth rate of fracture length (Fig. 7). Moreover, when reaching a displacement level of 20 m^3^/min, fractures display diminished width and height due to partial embedding within the isolation layer. While no substantial elevation in fracture height occurs at this point, indications suggest potential breakthrough across the isolation layer. Henceforth, it is recommended to uphold a controlled fracturing pumping displacement around approximately 18 m^3^/min.

### Optimization of fracturing scale

**Figure 8:**
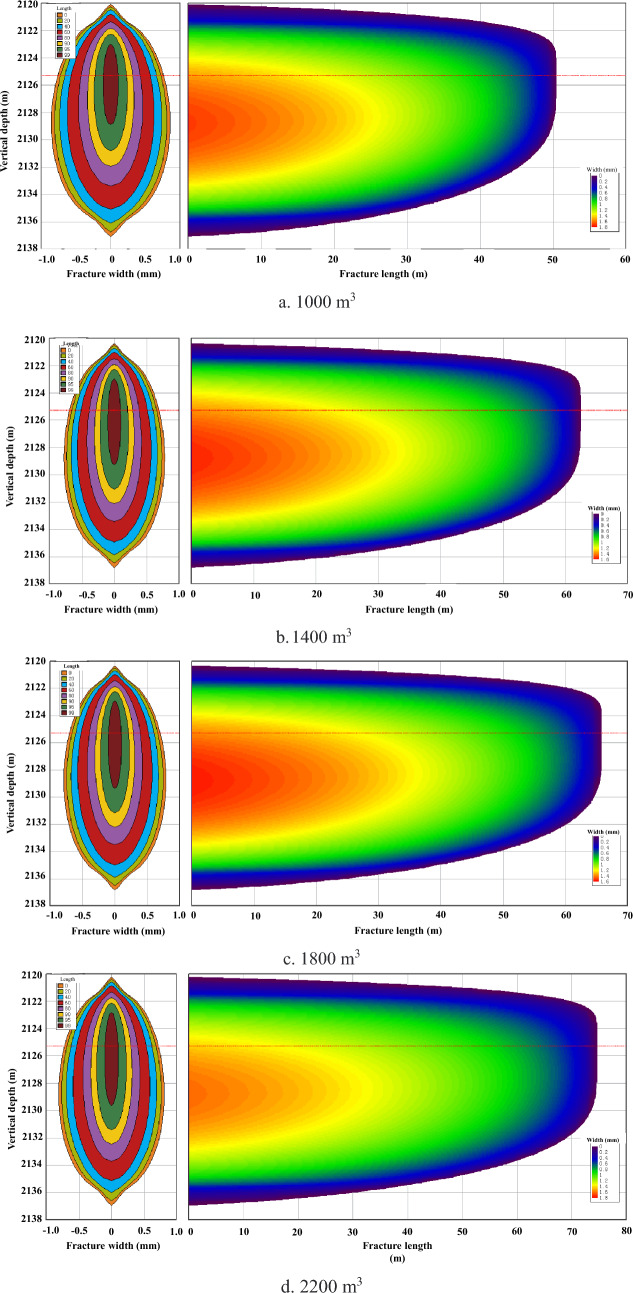
Fracture morphology under different injection volumes of fracturing fluid.

Considering the brittleness index characteristics of the reservoir, a qualitative analysis can be conducted on the amount of fracturing fluid, proppant concentration, and dosage within a certain range based on reference recommendations. Under identical conditions, larger fracturing scales result in greater fractures in each direction. However, cost factors must also be taken into account as increasing scale does not always lead to improved results due to gradual fracture propagation slowdown beyond a certain extent. Therefore, an optimal fracturing scale that meets layer-piercing requirements while effectively controlling costs exists. This section aims to determine critical and optimal fracturing scales for layer penetration through simulation calculations with volumes of 1000 m^3^, 1400 m^3^, 1800 m^3^ and 2200 m^3^ of fracturing fluid analyzed for their respective outcomes. The propagation morphology of fractures under different fracturing scales is illustrated in Fig. 8a–d.

**Figure 9:**
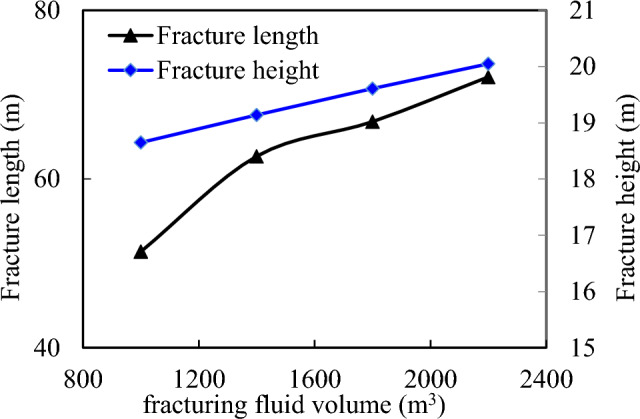
Influence of fluid volume on fracture morphology.

The analysis of Fig. 9 indicates that none of the calculation models exhibit any out-of-control fracture height after regulating the pumping displacement at 18 m^3^/min. However, a noticeable deceleration in the growth rate of fracture length becomes evident when increasing the pumping liquid volume to 1800 m^3^. Therefore, it is recommended to maintain control over the amount of fracturing fluid at approximately 1800 m^3^.

### Optimization of perforation method

The primary concern of fracture network fracturing lies in the ability of fractures to propagate and form a complex network, which is closely tied to the initiation of fractures at the perforation site. If the perforation coincides with the minimum fracture pressure, it will result in smaller branch angles. Conversely, if the perforation occurs at an angle relative to the minimum fracture pressure, fractures will initiate under higher pressure, leading to larger branch angles. Consequently, this can cause high frictional resistance and wellbore pressure buildup, setting the stage for subsequent fracture initiation. Prior to conducting reservoir fracturing operations, it is advisable to employ helical perforation techniques that facilitate multiple fracture initiations near the wellbore. In situations where unfavorable conditions exist at the perforation site, directional perforation perpendicular to the direction of maximum horizontal principal stress is recommended as it promotes branching fractures that extend towards the wellbore. This enhances complexity within the fracture network and improves overall effectiveness in constructing such networks.

**Figure 10:**
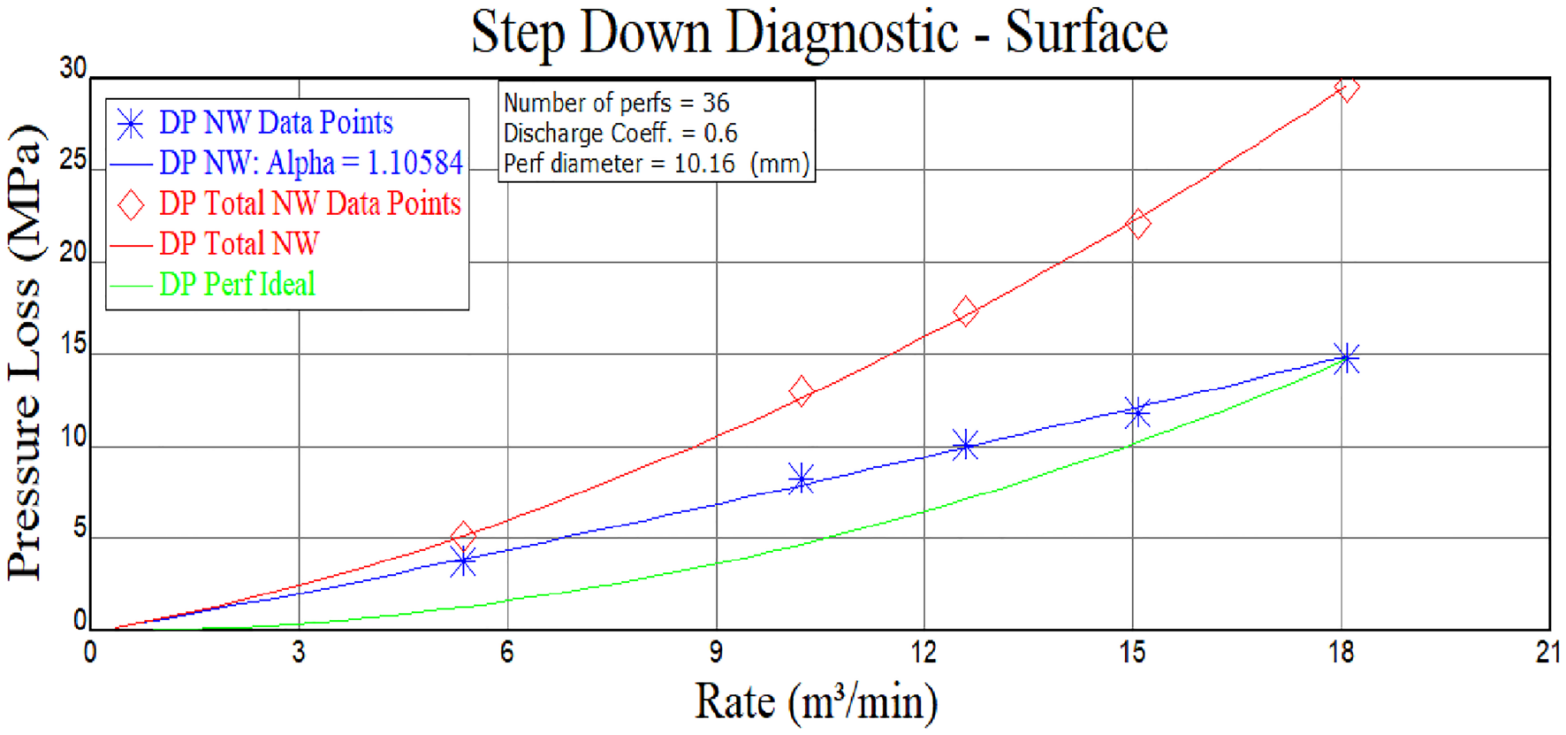
Stepped pumping displacement reduction analysis of Well A hole friction.

**Figure 11:**
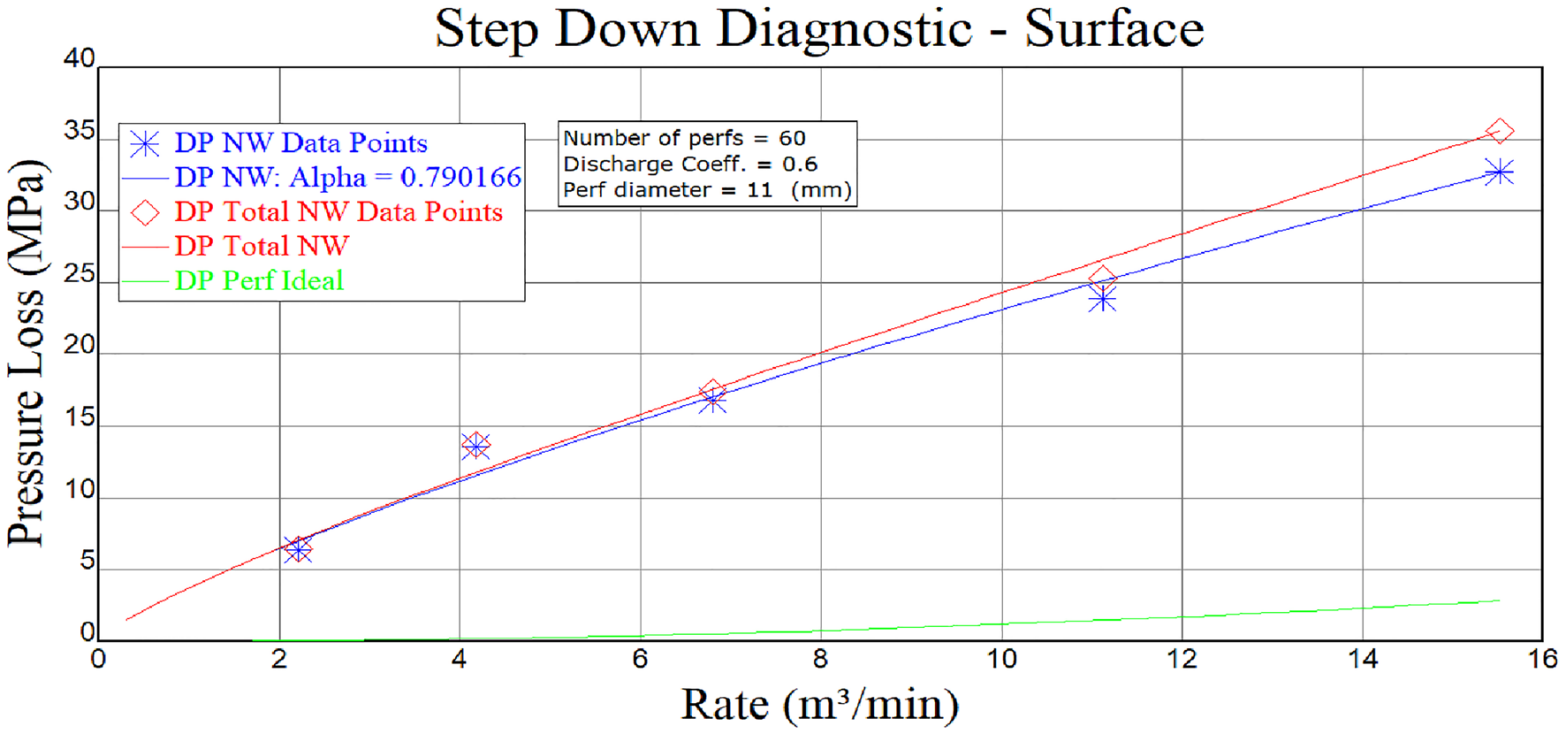
Stepped pumping displacement reduction analysis of Well B hole friction.

The net pressure serves as the driving force behind the expansion of artificial fractures. In cases where precise control over fracture height is desired, a higher net pressure facilitates the creation of longer and wider artificial fractures. However, both fluid flow within the fracture and local hydraulic energy losses contribute to a decrease in net pressure. Apart from utilizing effective drag reducing agents, there are no other viable methods to mitigate friction along the fracture surface. Nevertheless, hole friction can be adjusted through controllable engineering parameters such as perforation density and cluster arrangement. Therefore, we propose a method for optimizing the number of perforations by calculating hole friction. Calculation results indicate that a single section with 36 holes exhibits a hole friction of 14.6 MPa (Fig. 10). Conversely, nearby well B in the same block with 60 holes experiences only 4.0 MPa of hole friction (Fig. 11). This demonstrates that existing process measures and liquid systems are highly sensitive to changes in the number of perforations.

**Figure 12:**
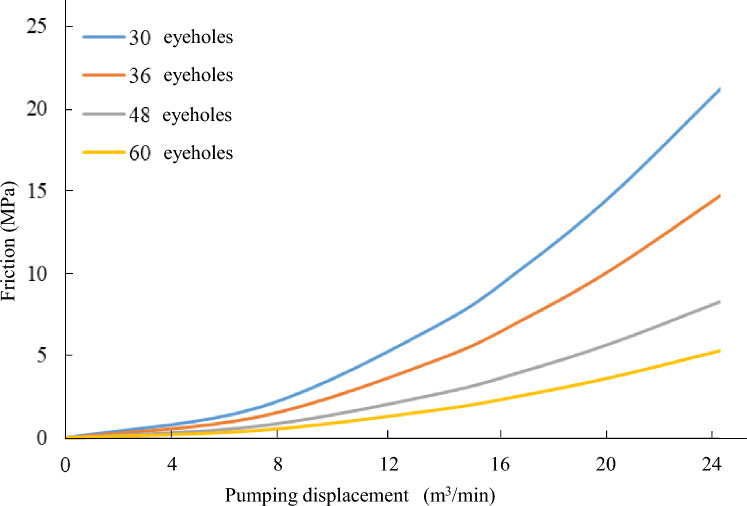
Corresponding chart of perforation number and hole friction.

According to the classical hydraulic formula, borehole friction is a critical parameter that must be taken into account during the drilling process due to its significant impact on operational efficiency and success. The magnitude of borehole friction is primarily influenced by the perforation number, which represents the number of holes drilled into the wellbore to facilitate fluid flow. To achieve more accurate calculations of borehole friction, it is essential to consider other factors such as drilling fluid properties, wellbore inclination, and drilling speed. In order to gain a more comprehensive understanding of the relationship between perforation number and borehole friction, we conducted an extensive calibration using measured data from wells A and B. This calibration process involved collecting and analyzing various drilling parameters including pressure drop, flow rate, and torque readings. These data were then utilized to generate corresponding charts (Fig. 12). These charts not only demonstrate a direct correlation between perforation number and borehole friction but also provide valuable insights into the drilling process for informed decision-making and improved efficiency.

## Conclusions

1. After considering factors such as rock brittleness index, natural fracture distribution, and rock mechanical parameters, an evaluation was conducted to assess the feasibility of implementing volume fracturing techniques on the reservoir. The results indicate that the average rock brittleness index of the reservoir is 63.25, with horizontal stress differences ranging from 3.5 to 8.7 MPa, predominantly below 10 MPa. Additionally, well-developed natural fractures are present, providing favorable geological conditions for the formation of a complex fracture network.
2. The distribution of stress and fracture pressure around the wellbore during near wellbore perforation completion was calculated, and it was predicted that the preferred location for fracturing fracture initiation was at the wellbore angle of 0° or 180°. By comparing with the actual construction well direction fracture pressure value, it is shown that the error range of the model in calculating fracture pressure is 2.37–7.93%, thereby confirming the reliability of the model.
3. It is recommended that reservoirs with a brittleness index of around 60 should utilize high liquid volume and large pumping displacement for fracturing. Conversely, reservoirs with lower brittleness index should decrease liquid dosage. Considering the extensive natural fracture development and significant fracturing fluid loss in the study area, it is advisable to employ a fracturing fluid scale exceeding 1800 m^3^ in the reservoir. The minimum required fracturing pumping displacement should be 18.0 m^3^/min, and each stage should have 36–48 perforation holes. Spiral perforation can be utilized as a technology to enhance multiple fractures in the wellbore due to minimal difference in horizontal stress within the reservoir.

## Data Availability

The datasets used and/or analyzed during the current study available from the corresponding author on reasonable request.
